# A Systematic Review on Antimicrobial Resistance in Ghana from a One Health Perspective

**DOI:** 10.3390/antibiotics13070662

**Published:** 2024-07-17

**Authors:** Eric S. Donkor, Alex Odoom, Abdul-Halim Osman, Samuel Darkwah, Fleischer C. N. Kotey

**Affiliations:** Department of Medical Microbiology, University of Ghana Medical School, Korle Bu, Accra P.O. Box KB 4236, Ghana; alexodoom2018@gmail.com (A.O.); abdulhalimosman20@gmail.com (A.-H.O.); kwekuadarkwah@gmail.com (S.D.); fleischarles@gmail.com (F.C.N.K.)

**Keywords:** One Health, human health, animal health, environment, antimicrobial resistance (AMR), multidrug resistance (MDR), surveillance, systematic review, Ghana

## Abstract

Background: Antimicrobial resistance (AMR) poses a global health threat, with lower-middle-income countries bearing a disproportionate burden. Surveillance of AMR under a One Health framework is needed to elucidate the associations among clinical, animal, and environmental AMR. This review aimed to describe the state of AMR in Ghana, focusing on One Health. Method: This review utilized the PRISMA guidelines and major databases to systematically search and analyze AMR in Ghana published from 1 January 2014 to 1 May 2023. Results: Out of the 48 articles that met the inclusion criteria, 28 studies were conducted on humans, 14 studies involved animals, and 6 studies focused on the environment. A total of 48 different pathogens were identified across the human, animal, and environmental sectors, with the most common being *Escherichia coli* (67%, *n* = 32), *Klebsiella* spp. (52%, *n* = 25), *Pseudomonas* spp. (40%, *n* = 19), and *Salmonella* spp. (38%, *n* = 18). Generally, a high prevalence of antibiotic resistance was observed among various bacterial species across the sectors. These bacteria exhibited resistance to commonly used antibiotics, with resistance to ampicillin and tetracycline exceeding 80%, and multidrug resistance (MDR) ranging from 17.6% in *Shigella* spp. to 100% in *Acinetobacter* spp. Conclusion: This review reaffirms the significant challenge of AMR in Ghana, with a high prevalence observed in the human, animal, and environmental sectors. Key pathogens (e.g., *Staphylococcus aureus* and *Escherichia coli*) found across the sectors emphasize the urgent need for a One Health approach to tackle AMR in Ghana.

## 1. Introduction

Recently, antimicrobial resistance (AMR) has been considered by the World Health Organization (WHO) as one of the top 10 global public health threats [1]. AMR limits the effectiveness of treatment options, leading to increased morbidity and treatment failure rates, longer hospital stays, elevated mortality rates, and higher healthcare costs [2]. In 2019, bacterial AMR directly caused 1.27 million deaths and contributed to 4.95 million deaths globally [3]. The economic costs of AMR are substantial, estimated to reach USD 1 trillion additional healthcare costs by 2050, along with significant GDP losses [4]. Although AMR affects countries across all regions and income levels, lower-middle-income countries are the most impacted [5]. In 2019, Ghana faced significant challenges related to AMR, with 5900 attributable deaths and an additional 25,300 associated deaths [6]. The country also ranked 36th globally in terms of age-standardized mortality rates associated with AMR per 100,000 population [6]. A major determinant of AMR and its spread is the weak enforcement and nonadherence to practice standards, policies, and regulations that govern the access to, and use of, antimicrobial agents in both humans and animals. In many sub-Saharan African countries such as Ghana, antimicrobial agents are easily obtained over the counter, facilitating their abuse [7,8]. While most high-income countries have adopted guidelines for the rational use of antibiotics, more efforts are needed in lower-middle-income countries [9,10,11].

Having recognized that the global pervasiveness of the AMR problem requires consolidated efforts for sustainable mitigation, the WHO Global Action Plan to address AMR emphasizes the application of the “One Health” approach [12]. This concept considers humans, animals, the environment, and the food chain as interconnected entities, owing to which resistance genes can be transferred from environmental bacteria to human pathogens [13]. Studying the interconnection among humans, animals, and the environment is crucial to preventing zoonotic disease outbreaks and maintaining ecosystem health, which impacts disease transmission and promotes a holistic approach to addressing health threats [14]. Surveillance of AMR under a One Health framework is needed to provide data for awareness and decision making and to enhance the understanding of the links between clinical, animal, and environmental AMR. In Ghana, a five-year National Action Plan (NAP) on AMR was instituted in 2017, focusing on enhanced surveillance through a One Health approach [15]. By monitoring the prevalence and distribution of resistant pathogens, the NAP can identify high-risk areas, track the movement of AMR, and develop targeted interventions to prevent further AMR spread. Although there has been a surge in AMR research data in Ghana, these data largely lack information on the interconnections among humans, animals, and the environment. This systematic review, therefore, examined AMR in Ghana from the One Health perspective, focusing on the antibiogram of bacterial pathogens.

## 2. Results

### 2.1. Search Results

**Figure 1:**
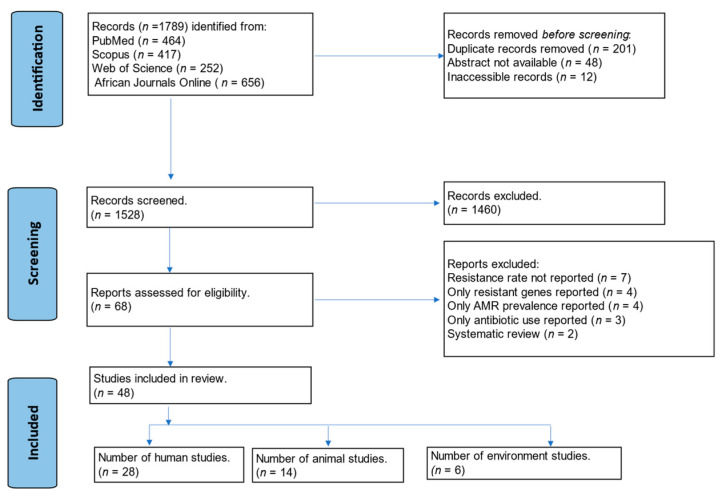
PRISMA flow diagram for the identification, screening, and evaluation of articles included in the study.

The initial search of the online databases identified a total of 1789 publications. After removing duplicates and inaccessible records, the titles and abstracts of the remaining 1528 records were screened. From these records, 1460 articles were excluded because they did not meet the inclusion criteria. Subsequently, 68 full-text articles were assessed for eligibility, of which 48 [16,17,18,19,20,21,22,23,24,25,26,27,28,29,30,31,32,33,34,35,36,37,38,39,40,41,42,43,44,45,46,47,48,49,50,51,52,53,54,55,56,57,58,59,60,61,62,63] met the inclusion criteria (Figure 1). These 48 articles included 28 studies on humans, 14 studies on animals, and six studies on the environment. The “human studies” included various populations, including healthy volunteers, patients, and food handlers. Studies involving animals included livestock such as fish, poultry, pigs, and cattle. The “environmental studies” included various samples: water and hospital surfaces (such as door handles and doctors’ mobile phones).

### 2.2. Study Characteristics

The descriptive features of the articles used in this systematic review are grouped into three sectors: “human studies”, “animal studies”, and “environmental studies” (Table S1). For the “human studies”, the majority (32.1%, *n* = 9) were conducted in the Greater Accra Region, followed by the Ashanti (18%, *n* = 5), Northern (11%, *n* = 3), Upper East (3.6%, *n* = 1), Western (7%, *n* = 2), Volta (7%, *n* = 2), and Central (3.6%, *n* = 1) Regions. Five studies were conducted at multiple centers across the Greater Accra, Eastern, Ashanti, and Volta Regions. Similarly, “animal studies” were conducted across various regions of Ghana, with the highest number of studies (35.7%, *n* = 5) in the Greater Accra Region, followed by the Ashanti Region (28.6%, *n* = 5). The Northern and Central Regions each had the least number of studies (7%, *n* = 1). Three studies were conducted at multiple centers across the Western, Central, Brong Ahafo, and Greater Accra Regions. Regarding “environmental studies”, the region with the most studies was the Greater Accra Region (50%, *n* = 3), followed by the Northern Region (33%, *n* = 2). One study was a multicenter study across the Greater Accra and Eastern Regions. Overall, the Greater Accra Region was the most frequent sampling location (35.4%, *n* = 17), followed by the Ashanti (18.6%, *n* = 9), Northern (13%, *n* = 6), Upper East (2%, *n* = 1), Volta (4%, *n* = 2), Western (4%, *n* = 2), and Central (4%, *n* = 2) Regions. Nine studies were conducted at multiple centers, while no studies were identified in the Upper West Region.

The studies analyzed in the review varied in design and included cross-sectional, longitudinal, and retrospective studies (Table S2). Among the “human studies”, the majority (39.3%, *n* = 11) were cross-sectional, followed by retrospective (18%, *n* = 5), retrospective cross-sectional (7.1%, *n* = 2), prospective (4%, *n* = 1), and prospective cross-sectional (4%, *n* = 1). For the “animal studies”, the majority (50%, *n* = 7) were cross-sectional. Similarly, for the “environmental studies”, the majority (33%, *n* = 2) were cross-sectional, followed by longitudinal (17%, *n* = 1). Overall, most studies used cross-sectional designs (41.7%, *n* = 20), followed by retrospective (10%, *n* = 5), retrospective cross-sectional (4.2%, *n* = 2), prospective (2.1%, *n* = 1), prospective cross-sectional (2.1%, *n* = 1), and longitudinal (2.1%, *n* = 1) designs. However, 18 studies (37.5%) did not specify the design used.

Most of the “animal studies” (50%, *n* = 7) were conducted on chicken/poultry, followed by cattle (36%, *n* = 5), fish (14%, *n* = 2), and pigs (7%, *n* = 1) (Table S2). Information on the common infections investigated in the “human studies” was included. The majority (14%, *n* = 4) were conducted on patients with urinary tract infection (UTI), followed by those with bloodstream infections (11%, *n* = 3), wound infection (7%, *n* = 2), cholera (7%, *n* = 2), HIV (4%, *n* = 1), diarrhea (4%, *n* = 1), and sepsis (4%, *n* = 1). Information on the specimen types used in the studies was also included. For samples reported from “human studies”, the most abundant was urine (46%, *n* = 13), followed by blood (43%, *n* = 12), wound swabs (36%, *n* = 10), high vaginal swabs (21%, *n* = 6), ear swabs (18%, *n* = 5), urethral swabs (14%, *n* = 4), stool (14%, *n* = 4), sputum (11%, *n* = 3), aspirates (11%, *n* = 3), nasopharyngeal swabs (11%, *n* = 3), semen (7%, *n* = 2), cerebrospinal fluid (7%, *n* = 2), fecal sludge (4%, *n* = 1), and palm swabs (4%, *n* = 1). Samples from “animal studies” were obtained from meat (36%, *n* = 5), feces (36%, *n* = 5), intestines (7%, *n* = 1), poultry feed (7%, *n* = 1), poultry drinking water (7%, *n* = 1), and rectal swabs (7%, *n* = 1). Samples from the environmental sector were obtained from water sources (67%, *n* = 4), mobile phones (17%, *n* = 1), and door handles (17%, *n* = 1).

The identification methods utilized in these studies included culture-based techniques (92.9%, *n* = 26), standard biochemical tests (78.6%, *n* = 22), PCR (10.7%, *n* = 3), and MALDI-TOF assays (25%, *n* = 7). In terms of susceptibility testing, the Kirby-Bauer disk diffusion method was the most commonly used method (75%, *n* = 21), while a few studies (14.3%, *n* = 4) employed the VITEK 2 system. Additionally, most studies (71.4%, *n* = 20) adopted the CLSI and EUCAST (17.9%, *n* = 5) guidelines (Table S3).

### 2.3. Bacterial Agents and Antibiotic Resistance

**Figure 2:**
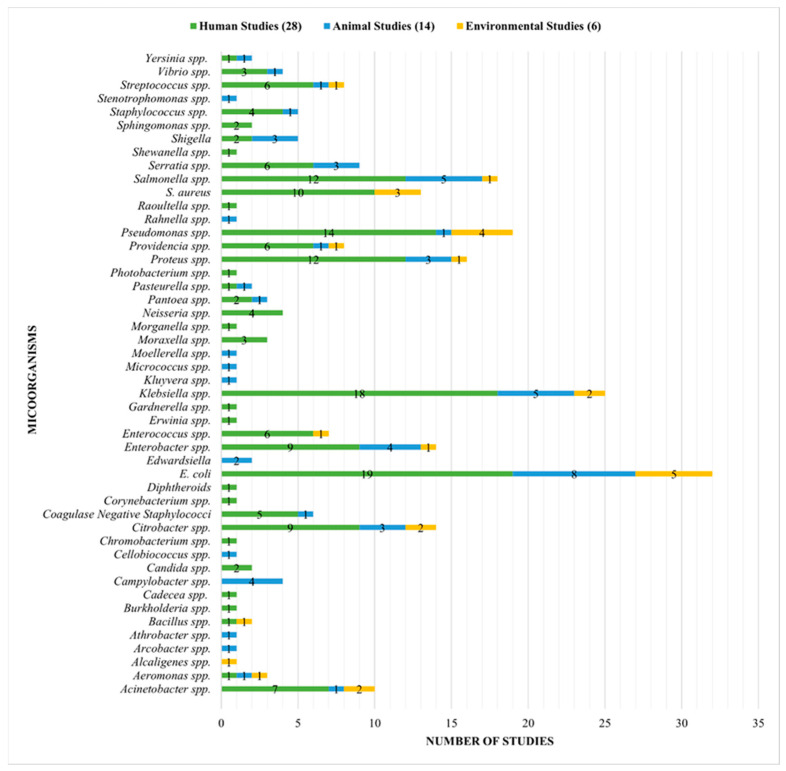
List of all bacteria isolated across the studies.

Forty-eight different bacterial agents were isolated across the “human”, “animal” and “environmental” sectors, with approximately one-third belonging to the Enterobacteriaceae family (Figure 2). *E. coli* was the most commonly reported organism for each of the three sectors: 19 “human studies”, eight “animal studies”, and five “environmental studies”. *Klebsiella* spp. was the next most commonly reported organism for each of the three sectors: 18 “human studies”, five “animal studies” and two “environmental studies”.

A high prevalence of resistance was observed among various bacterial species isolated from humans (Table 1). For instance, the prevalence of resistance in *Acinetobacter* spp. ranged from 35% to 67% for ciprofloxacin, tetracycline, gentamicin, and trimethoprim-sulfamethoxazole, while the prevalence of resistance to ampicillin and ceftriaxone was >80%. *Citrobacter* spp. exhibited resistance prevalence ranging from 22.1% to 93% for ciprofloxacin, tetracycline, gentamicin, trimethoprim-sulfamethoxazole, and ceftriaxone. *Enterococcus* showed high resistance prevalence of 100% to tetracycline [25,31], and trimethoprim-sulfamethoxazole [31]. Similarly, a high prevalence of resistance was observed among bacterial agents isolated from animals, as well as from the environment. For example, in the case of *Campylobacter* spp., which were reported from only animals, one study reported a resistance prevalence of 75% to ciprofloxacin and 70% to tetracycline [55], while another reported a resistance prevalence of 44% to ciprofloxacin, 81% to tetracycline, 81% to ampicillin, 56% to trimethoprim-sulfamethoxazole and 88% to chloramphenicol [52]. The prevalence of resistance to several antibiotics, including ciprofloxacin, ampicillin, trimethoprim-sulfamethoxazole, and ceftriaxone, was 100% in *Pseudomonas* spp. isolated from the environment [60].

Multidrug resistance, generally defined as the ability of bacteria to resist three or more antibiotics or three or more classes of antimicrobial drugs, was commonly observed in “human”, “animal” and “environmental” studies across a wide spectrum of bacterial pathogens. For the “human studies”, two reported MDR in *S. aureus* at a prevalence of 35.7% and 84.6% [16,20]. Two others reported MDR in *Salmonella* spp. at a prevalence of 41.5% and 99.6% [39,62]. In six “human studies”, *E. coli* exhibited MDR, with the prevalence ranging from 41.5% to 99.6% [18,23,27,34,36,41]. MDR prevalence of 17.6%, 78.4%, 88%, 89.5% and 100% were reported for *Shigella* spp. [17], *Vibrio cholerae* [26], *K. pneumoniae* [35], *P. aeruginosa* [28], and *A. baumannii* [30], respectively. Six “animal studies” reported MDR prevalence ranging from 14.9% to 100% in *E. coli* [45,46,48,49,51,63]. Four “animal studies” reported MDR traits ranging from 20% to 66.6% in *Campylobacter* spp. [50,52,53,55]. One animal study reported an MDR prevalence of 40.4% in *Salmonella* spp. [32], while another reported an MDR prevalence of 19.1% in coagulase-negative Staphylococci [47]. Two “environmental studies” reported MDR in *S. aureus* at a prevalence of 19% and 59.6% [57,61], while two others reported MDR in *E. coli* at 28% and 58.3% prevalence [59,60].

### 2.4. Risk of Bias

**Figure 3:**
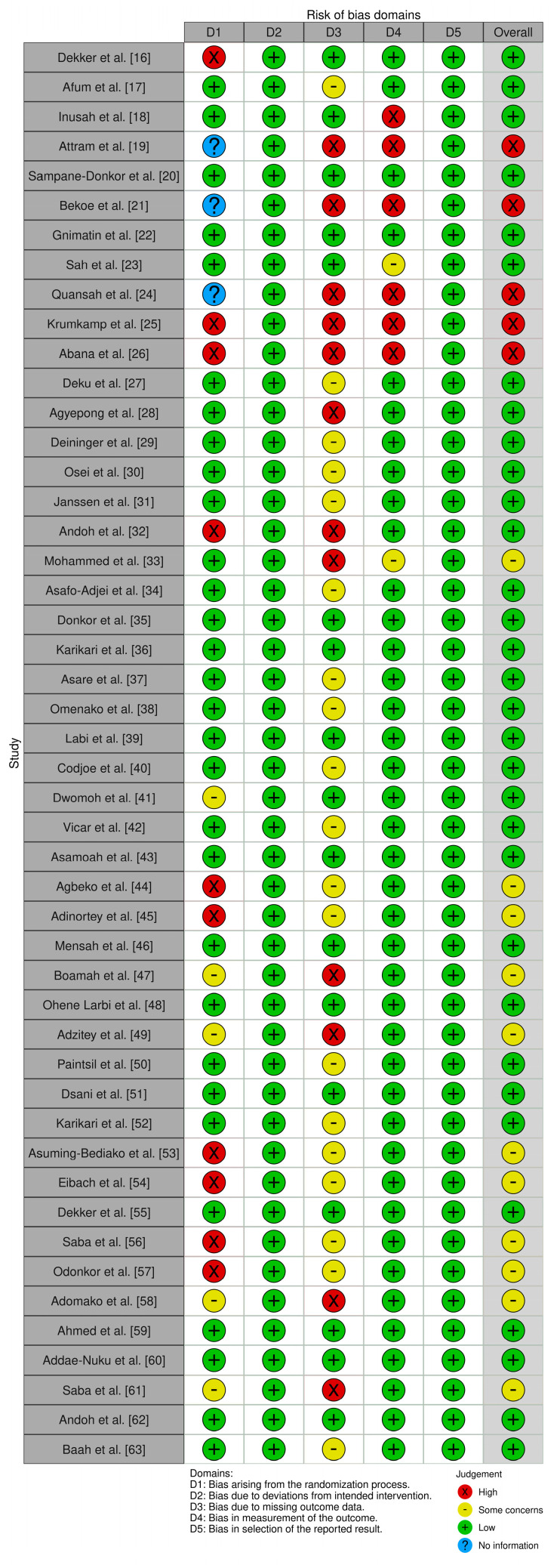
Assessment of bias of the included studies using the Cochrane risk-of-bias tool (ROB2) [16,17,18,19,20,21,22,23,24,25,26,27,28,29,30,31,32,33,34,35,36,37,38,39,40,41,42,43,44,45,46,47,48,49,50,51,52,53,54,55,56,57,58,59,60,61,62,63].

The risk of bias assessment in Figure 3 provides a comprehensive evaluation of the 48 studies included in this systematic review. The Robvis tool used for evaluation categorizes the risk of bias into three levels: low risk (shown in green), some concerns (in yellow), and high risk (in red). The overall low risk of bias across all evaluated domains indicates that the studies are methodologically sound and robust. 

## 3. Discussion

Antimicrobial resistance is a significant global health threat that affects human health, as well as animal and environmental health [64]. The One Health approach emphasizes the importance of monitoring and surveillance systems that combine data from human, animal, and environmental sources [13,65]. Thus, to gain a comprehensive understanding of the AMR situation in Ghana, this systematic review employed a One Health approach. Greater Accra Region was the most frequently sampled location for “human”, “animal”, and “environmental” studies, likely due to its high population density and industrial activities. Furthermore, this could be attributed to the presence of numerous research institutions, academic centers, and tertiary hospitals in the region. Interestingly, the Ashanti Region had more animal research than clinical research, which may be due to the high prevalence of agricultural activities in the region. It is essential to note that the Central, Volta, Western, Upper East, and Eastern Regions had fewer studies conducted across all sectors, which could be due to various factors, such as inadequate research infrastructure and funding. There is an urgent need for increased investment in research and surveillance programs, particularly in regions of the country that have received less focus regarding AMR surveillance efforts.

A decade ago, neglected tropical diseases, malaria, HIV/AIDS, sexually transmitted infections, and other infectious conditions in Ghana had higher mortality rates compared to AMR-related deaths [6]. However, presently, AMR-related deaths in Ghana have significantly increased, surpassing deaths from all the aforementioned diseases [6]. This rise can be attributed to resistant pathogens, as evident from this systematic review. The results indicate that AMR is a major concern across multiple bacterial species in Ghana; there are significant levels of resistance in bacteria commonly associated with human infections, such as *E. coli*, *Klebsiella* spp., *S. aureus*, and *Salmonella* spp. Notably, the presence of MDR bacteria (that is bacteria exhibiting resistance to multiple antibiotics) is particularly alarming. MDR was commonly observed among members of the Enterobacteriaceae, including *E. coli* and *K. pneumoniae*, which can cause severe infections and have multiple resistance mechanisms, such as extended-spectrum beta-lactamase (ESBL) and carbapenemase production [28,66,67]. The high prevalence of MDR-Enterobacteriaceae in animals can pose a risk to human public health, both directly and indirectly. *E. coli*, a common colonizer of the gastrointestinal tract in humans and animals [68,69], was the most studied bacterial species, followed by species of *Klebsiella*, *Pseudomonas*, and *Salmonella*. *Klebsiella pneumoniae* is naturally present in the respiratory and gastrointestinal tracts of humans and has a lower occurrence in animals, possibly due to diet, competition with other bacteria, or genetic virulence factors [70,71,72]. *Pseudomonas* spp., on the other hand, typically take advantage of weakened immune systems and causes infections that can be acquired in hospitals or the community, affecting both humans and animals [73]. *Salmonella* spp. are foodborne pathogens that cause gastroenteritis and other serious illnesses in humans and animals [74]. Given the ability of these pathogens to infect multiple host types, it is crucial to adopt a One Health approach that recognizes the interdependence of human, animal, and environmental health in addressing antibiotic resistance.

MDR bacteria can be transmitted from animals to humans through direct contact or the consumption of contaminated food products [75]. Studies have revealed the presence of MDR bacteria, including methicillin-resistant *S. aureus* (MRSA), ESBL-producing Enterobacteriaceae, and MDR-*Salmonella*, in meat products [63,76,77,78,79]. Similarly, a recent study in Romania found that pigs, and to some extent cattle, serve as significant natural reservoirs for zoonotic MDR *Campylobacter* strains [80]. However, in Ghana, AMR surveillance has mostly overlooked animal-derived foods, which are a significant source of antibiotic-resistant zoonotic pathogens. Monitoring AMR in animal-derived foods is just as essential as monitoring it in humans. The diverse range of bacterial contaminants found reflects the various infections that consumers may be exposed to, particularly if the meat is not cooked thoroughly before consumption, leading to foodborne illnesses and the transmission of zoonotic diseases. The involvement of MDR organisms in meat contamination could exacerbate the already severe problem of AMR, which is projected to cause 10 million deaths annually, amidst other adverse impacts [81]. One potential approach to broaden AMR surveillance is to incorporate wastewater as a significant reservoir of MDR pathogens. Although there is mounting evidence of bacterial resistance to antibiotics in Ghana, the available data primarily originate from clinical samples, with limited information on environmental AMR. The environmental dimension of AMR is closely linked to the use and disposal of antibiotics in various sectors. For instance, animal husbandry practices mainly utilize tetracyclines, penicillin, streptomycin, and ciprofloxacin as prophylaxes [82,83]. The release of antibiotic residues and resistant bacteria into the environment through wastewater effluents or agricultural runoffs contributes to the dissemination of resistance genes in environmental bacteria [84]. This environmental reservoir of AMR genes can potentially spread to human and animal pathogens, further exacerbating this problem. Raising awareness about AMR across sectors (human health, animal health, and the environment) can promote responsible antibiotic use and infection prevention, ultimately contributing to collective efforts in combating AMR.

## 4. Methods

### 4.1. Preferred Reporting Items for Systematic Reviews (PRISMA) Guidelines

We followed the Preferred Reporting Items for Systematic Reviews and Meta-Analyses (PRISMA) guidelines [85] to ensure a systematic and transparent approach to our literature search and review. The PRISMA guidelines provide a comprehensive checklist and flow diagram for record identification, screening, and evaluation.

### 4.2. Search Strategy

A literature search was conducted using various databases and indexing services (PubMed, Scopus, Web of Science and African Journals Online) to identify all published studies on AMR in Ghana. We searched for English-language articles published between 1 January 2014, and 1 May 2023, as most relevant studies were published during that period. Additionally, we checked the reference lists of relevant articles for additional studies to include in our review. The search terms used included “antimicrobial resistance”, “antibiotic resistance”, “multidrug resistant bacteria”, and “Ghana”. A combination of keywords and MeSH terms was used to ensure that the search was comprehensive and specific to our research question. Boolean operators such as “AND” and “OR” were used to combine search terms and increase the search sensitivity. The PubMed, Scopus, Web of Science and African Journals Online databases were chosen for the search because they are widely used and have broad coverage of scientific publications. The search was not limited to any specific study design, population group, or outcome measure to ensure the capture of all relevant studies.

### 4.3. Inclusion and Exclusion Criteria

A two-step screening process was employed to identify relevant studies for the systematic review. First, the titles and abstracts of all studies identified were screened to exclude any irrelevant or duplicate studies. Second, we assessed the full texts of the remaining studies to determine their eligibility for inclusion in the review. We employed predefined inclusion and exclusion criteria to evaluate the studies. Studies reporting antibiotic resistance in bacteria from humans, animals, and the environment in Ghana were considered eligible. We excluded studies that focused solely on resistance genes without reporting phenotypic antibiogram data. Additionally, studies that reported only antibiotic use and those that were systematic reviews were excluded. Additionally, studies published before 2014 and those not conducted in Ghana were excluded. Two independent reviewers performed the screening process with fixed inclusion criteria. Mendeley Desktop version 1.19.8 was used to manage the search results and identify any duplicate records from the databases.

### 4.4. Data Extraction and Analysis

Microsoft Excel 365 software was used to manage the data from the studies reviewed. Two authors worked independently using a data abstraction format prepared in Microsoft Excel 365 to extract data from the studies. The extracted information included author(s), year of publication, geographical area of the study, study design, sector of study (human, animal, and the environment), specimen type, bacteria isolated, antibiotics tested, and antibiotic resistance data. Microsoft Excel 365 was used for all computations and data visualization. The frequencies and prevalence of bacterial agents, including their antibiogram, resistance and multidrug resistance, were calculated; comparisons of these data were made across the different sectors of humans, animals and the environment. A statistical significance threshold was set at *p* < 0.05. To measure the variability between studies, the I^2^ statistic and Cochran’s Q test were used, with cutoff values of 25%, 50%, and 75% indicating low, moderate, and high heterogeneity, respectively [86]. The results are presented in tables, text, and figures.

### 4.5. Evaluation of Bias

The Cochrane risk-of-bias tool (ROB2) [87] was used to assess the risk of bias in each study, and the results were visualized using the Robvis tool [88]. The Robvis tool evaluates five domains of bias: randomization, deviations from intended interventions, missing outcome data, measurement of outcomes, and selection of reported results. Each domain was classified as low risk, high risk, or some concerns, and studies were categorized as low risk if all domains showed low risk, high risk if any domain showed high risk, or some concerns if there were concerns in at least one domain. 

## 5. Conclusions

This comprehensive review underscores the considerable burden of AMR in Ghana, with a high prevalence observed across the human, animal, and environmental sectors. This study revealed alarming levels of resistance among key pathogens, particularly the widespread resistance to multiple antibiotics among *S. aureus* and *E. coli* across various sectors. These findings emphasize the urgent need for a One Health approach for addressing AMR in Ghana.

## Figures and Tables

**Table 1:** Antibiotic resistance profile of commonly isolated bacteria across sectors.

Bacteria Isolates	CIP			TET			GEN			AMP			SXT			CTX			CHL			References
	Hu	An	En	Hu	An	En	Hu	An	En	Hu	An	En	Hu	An	En	Hu	An	En	Hu	An	En	
*Acinetobacter* spp.	35–44	-	-	55	-	-	37–63	-	-	95	-	-	58–67	-	-	88–89	-	-	-	-	-	[22,31,35]
*Aeromonas* spp.	-	-	13	-	-	23	-	-	17	-	-	-	-	-	-	-	-	-	-	-	-	[58]
*Campylobacter* spp.	-	44–75	-	-	70–100	-	-	-	-	-	81–96	-	-	56	-	-	-	-	-	88	-	[50,53,55]
*Citrobacter* spp.	22–80	-	100	78–93	53	-	22.2–66.7	5	97	-	100	100	50	-	100	78	45	-	-	58	-	[18,23,29,34,44,60]
*E. coli*	46–89	2–54	6–17	25–92	45–100	37	17–62	0–39	3.7–27	88–100	100	100	69–92	8–21	66–100	49- 78	0–17	34–100	9–83	0–46	-	[17,18,22,23,25,29,31,34,35,37,41,43,44,46,48,49,51,56,58,59,60]
*Enterobacter* spp.	11–73	-	-	87–100	82	-	25.9–47	0	-	100	100	-	37–68	-	-	52–68	45	-	-	36	-	[22,23,31,34,44]
*Enterococcus* spp.	-	-	-	100	-	-	-	-	-	0	-	-	58–100	-	-	-	-	-	44	-	-	[25,31]
*Klebsiella* spp.	14–76	-	12	71–89	55	-	29–83	14	0	100	100	100	48–95	-	46	64–91	36	-	50–92	55	-	[18,22,23,24,25,31,34,35,41,43,44,60]
*Moraxella* spp.	69	-	-	50–90	-	-	28	-	-	-	-	-	67	-	-	-	-	-	-	-	-	[18,22]
*Neisseria gonorrhoeae*	82	-	-	100	-	-	-	-	-	-	-	-	-	-	-	-	-	-	-	-	-	[19]
*Proteus* spp.	5–100	-	-	80–100	50	-	5–100	0	-	70–90	100	-	43–75	-	-	-	50	-	64	0	-	[18,25,29,31,34,44]
*Pseudomonas* spp.	15–100	-	5–100	80–100	-	-	10–40	-	11–100	96	-	100	92	-	100	-	-	-	-	-	-	[18,22,23,29,31,34,35,59,60]
*Staphylococcus* spp.	0–71	-	5–9	57–100	24	28–64	4–67	0	9	-	100	13	32–100	-	11	-	-	-	60–71	-	81.8	[16,18,21,29,34,44,56,61]
*Salmonella* spp.	0–67	63–65	-	35	60–100	-	0–67	0	-	33–57	26–80	-	17–66	69	-	0–22	20	-	34	6–20	-	[17,23,32,34,44]
*Serratia* spp.	-	-	-	>90	-	-	-	-	-	>90	-	-	-	-	-	>90	-	-	>90	-	-	[18]
*Shigella* spp.	-	-	-	90–100	67–100	-	-	0	-	-	98–100	-	39	-	-	-	100	-	-	100	-	[17,18,44,45]
*Streptococcus* spp.	-	-	-	-	17	-	-	17	-	100	83	-	-	-	-	-	-	-	63	-	-	[25,44]
*Vibrio* spp.	25–90	-	-	65	-	-	-	-	-	-	-	-	38	-	-	83.3	-	-	50	-	-	[17,26]

CHL: chloramphenicol, CTX: ceftriaxone, SXT: trimethoprim-sulfamethoxazole, AMP: ampicillin, GEN: gentamicin, TET: tetracycline, CIP: ciprofloxacin. Hu: “human studies”, An: “animal studies”, En: “environmental studies”, and -: no data available.

## Data Availability

Available upon request.

## Supplementary Materials

The following supporting information can be downloaded: https://www.mdpi.com/article/10.3390/antibiotics13070662/s1, Figure S1: Antibiotics used in the studies; Table S1: List of full-text articles included in the systematic review; Table S2: Demographic features of studies included in the systematic review; Table S3: Characteristics of studies included in the systematic review; Table S4: Medically important bacteria isolated in the studies.
