# Supplementary material for: A Systematic Review on Antimicrobial Resistance in Ghana from a One Health Perspective
Source: Antibiotics (Basel). 2024 Jul 17;13(7):662. doi: 10.3390/antibiotics13070662 (PMC11274323; doi:10.3390/antibiotics13070662)
Supplement: Supplementary file 1 [file antibiotics-13-00662-s001.zip › antibiotics-3049339-supplementary.pdf]

Supplementary result

Table S1: List of full-text articles included in the systematic review.

Type of Study	Authors	Year published	Region
**Human**	Dekker et al.[1]	2016	Ashanti
	Afum et al. [2]	2022	Greater Accra
	Inusah et al. [3]	2021	Upper East
	Attram et al. [4]	2019	Multicenter
	Sampane-Donkor et al. [5]	2017	Greater Accra
	Bekoe et al. [6]	2022	Greater Accra
	Gnimatin et al. [7]	2022	Northern
	Sah, & Feglo. [8]	2022	Western
	Quansah et al. [9]	2019	Multicenter
	Krumkamp et al. [10]	2020	Ashanti
	Abana et al. [11]	2019	Greater Accra
	Deku et al. [12]	2022	Volta
	Agyepong et al. [13]	2018	Ashanti
	Deininger et al. [14]	2022	Volta
	Osei et al. [15]	2022	Greater Accra
	Janssen et al. [16]	2018	Western
	Andoh et al. [17]	2017	Ashanti
	Mohammed et al. [18]	2018	Greater Accra
	Asafo-Adjei et al. [19]	2018	Greater Accra
	Donkor et al. [20]	2023	Multicenter
	Karikari et al. [21]	2022	Northern
	Asare et al. [22]	2022	Central
	Omenako et al. [23]	2022	Ashanti
	Labi et al. [24]	2014	Greater Accra

	Codjoe et al. [25]	2019	Multicenter
	Dwomoh et al. [26]	2022	Greater Accra
	Vicar et al. [27]	2023	Northern
	Asamoah et al. [28]	2022	Multicenter
**Animal**	Agbeko et al. [29]	2022	Multicenter
	Adinortey et al. [30]	2020	Central
	Mensah et al. [31]	2022	Greater Accra
	Boamah et al. [32]	2017	Multicenter
	Ohene Larbi et al. [33]	2022	Greater Accra
	Andoh et al. [34]	2016	Multicenter
	Adzitey et al. [35]	2020	Northern
	Paintsil et al. [36]	2022	Ashanti
	Dsani et al. [37]	2020	Greater Accra
	Karikari et al. [38]	2017	Ashanti
	Asuming-Bediako et al. [39]	2022	Greater Accra
	Baah et al. [40]	2022	Greater Accra
	Eibach et al. [41]	2018	Ashanti
	Dekker et al. [42]	2019	Ashanti
**Environmental**	Saba et al. [43]	2022	Northern
	Odonkor et al. [44]	2022	Multicenter
	Adomako et al. [45]	2021	Greater Accra
	Saba et al. [46]	2017	Northern
	Ahmed et al. [47]	2022	Greater Accra
	Addae-Nuku et al. [48]	2022	Greater Accra

Table S2: Demographic features of studies included in the systematic review.

**Demographic Features**	**Human Studies (28) n (%)**	**Animal Studies (14) n (%)**	**Environmental Studies (6) n (%)**	**References**
**Common Infection**				
Blood stream infection	3 (10.71)			[1,20,24]
Cholera	2 (7.14)			[2,11]
HIV	1 (3.57)			[5]
Infected wound	2 (7.14)			[10,16]
Septic/Sepsis	1 (3.57)			[23]
UTI	4 (14.29)			[19,21,22,28]
Diarrheal	1 (3.57)			[2]
**Animal Study**				
Fish		2 (14.29)		[29,30]
Chicken/Poultry		7 (50)		[31,32,34–36,39,41]
Pigs		1 (7.14)		[33]
Cattle		5 (35.71)		[35–38,40]
**Human Study**				
Patients	15 (53.57)			[1,2,4,7,10,12–14,16,17,19–21,24,26]
Children	2 (7.14)			[5,15]
Neonates	1 (3.57)			[23]
Healthy Volunteer	1 (3.57)			[6]
Food Handlers	1 (3.57)			[27]

**Specimen type**				
Intestinal		1 (7.14)		[29]
Fecal sample		5 (35.71)		[31,32,34,36,38]
Rectal swab		1 (7.14)		[33]
Poultry drinking water		1 (7.14)		[34]
Poultry feed		1 (7.14)		[34]
Meat		5 (35.71)		[35,37,39,40,42]
Blood	12 (42.86)			[3,7–9,12,13,17,18,20,22–24]
Stool	4 (14.29)			[2,17,22,26]
Aspirate	3 (10.71)			[3,9,26]
Cerebrospinal fluid	2 (7.14)			[3,22]
Ear swab	5 (17.86)			[3,9,12,18,22]
High Vaginal swab	6 (21.43)			[3,7,9,12,18,22]
Semen	2 (7.14)			[3,18]
Sputum	3 (10.71)			[3,7,9]
Urethral swab	4 (14.29)			[3,4,18,22]
Wound swab	10 (35.71)			[3,7–10,13,16,18,22,26]
Urine	13 (46.43)			[3,6–9,12–14,18,19,21,22,26]
Nasopharyngeal swab	3 (10.71)			[3,5,15]
Fecal sludge	1 (3.57)			[17]

Palm swab	1 (3.57)			[27]
Mobile phone			1 (16.67)	[43]
Water source			4 (66.67)	[44,45,47,48]
Door handles			1 (16.67)	[46]

Table S3: Characteristics of studies included in the systematic review.

Author	Region	Year	Study Design	Study Period	Definition of MDR Adopted	Microorganisms studied	Main MDR Microorganism	No. of Isolate	No. of MDR (%)	Identification of microbes	AST Testing Method	Break point Used for MIC	Sample Features	Av. age	Subjects (N)	Settings	Sector
Afum et al. [2]	Greater Accra	2022	Cross-sectional	Aug 2017-May 2021	Resistant to ≥ 3 classes of antibiotics	*Escherichia coli; Salmonella* spp; *Vibrio* spp*;* and *Shigella* spp.	*Shigella* spp.	772	17.6%	Culture-based; standard biochemical tests; MALDI-TOF assay; and serological analysis	Kirby-Bauer disk diffusion and Microscan autoScan4 MIC panels	CLSI	Diarrhea Patients	29.3	792	Five health facilities	Human
Sampane-Donkor et al. [5]	Greater Accra	2017	Cross-sectional	Feb-Apr 2015	Resistant to ≥ 3 classes of antibiotics	*Staphylococcus aureus*; *Moraxella catarrhalis*; *Streptococcus pneumoniae; Coagulase-Negative Staphylococci; Diphtheroid; Streptococcus viridans; Neisseria meningitidis;* and *Citrobacter* spp.	*Staphylococcus aureus*	221	84.6%	Culture-based; and standard biochemical tests	Kirby-Bauer disk diffusion	CLSI	HIV infected children	5.8	118	Korle-Bu Teaching Hospital	Human
Deku et al. [12]	Volta	2021	Cross-sectional	Apr 2018-Apr 2020	n.a	Extended-spectrum b-lactamase	*Escherichia coli*	135	41.5%	Culture-based; standard biochemical tests; and PCR	Kirby-Bauer disk diffusion	CLSI	General population	33	135	Ho teaching hospital laboratory	Human
Osei et al. [15]	Greater Accra	2022	Cross-sectional	Sep-Dec 2016	n.a	Aerobic GNB	*Acinetobacter baumannii*	57	100%	Bruker BD MALDI-TOF MS	Kirby-Bauer disk diffusion	CLSI	Under-Five children in nurseries and kindergartens	39 months	410	Nurseries and kindergartens	Human
Asafo-Adjei et al. [19]	Greater Accra	2018	Cross-sectional	Jan-Jun 2017	n.a	*Escherichia coli; Klebsiella* spp.; *Citrobacter* spp.; *Enterobacter* spp.;	*Escherichia coli*	144	98.1%	Culture-based; and standard biochemical tests	Kirby-Bauer disk diffusion	CLSI	Bladder outlet obstruction	69.1	188	Korle-Bu Teaching Hospital	Human

						Pseudomonas spp.; *Proteus* spp.; *Providencia* spp.; *Serratia* spp.; *Salmonella* spp.; *Staphylococcus aureus*; Coagulase-Negative *Staphylococcus aureus*; and *Shigella* spp.							patients				
Donkor et al. [20]	Multicenter (Accra, Eastern, Ashanti, and Volta)	2023	Cross-sectional	Apr-Dec 2021	In vitro non-susceptibility to ≥1 agent in ≥3 antimicrobial classes	*Acinetobacter baumannii; Escherichia coli; Klebsiella pneumoniae; Pseudomonas aeruginosa;* and *Salmonella* spp.	*Klebsiella pneumoniae*	334	88%	BD Bruker IVD MALDI-TOF version 4.2.100	BD Phoenix M50 analyzer and Kirby-Bauer disk diffusion	CLSI	Patients with bloodstream infections	6	334	Hospitals	Human
Omenako et al. [23]	Ashanti	2022	Cross-sectional	Jan-Dec 2021	n.a	*Coagulase-negative Staphylococcus; Staphylococcus aureus; Enterococcus* spp.; *Streptococcus* spp.; *Pseudomonas* spp.; *Klebsiella* spp.; and *Escherichia coli*		70		Culture-based	Kirby–Bauer disc diffusion	CLSI	Neonates Admitted with Suspected Sepsis	1 day	549	Komfo Anokye Teaching Hospital (KATH)	Human
Dwomoh et al. [26]	Greater Accra	2022	Cross-sectional	Dec 2020-Mar 2021	Resistant to at least one agent belonging to three or more antibiotic classes	*Escherichia coli;* and *Klebsiella pneumoniae*	*Escherichia coli*	144	69.4%	Culture-based; and standard biochemical tests	Kirby–Bauer disc diffusion	CLSI	Clinical isolate		144	Korle Bu Teaching Hospital (KBTH).	Human
Vicar et al. [27]	Northern	2023	Cross-sectional	Nov 2020-Mar 2021	n.a	*Escherichia coli*; *Klebsiella* spp.; Citrobacter spp.; *Proteus* spp.; Enterobacter spp.; *Pseudomonas aeruginosa;* Coagulase *negative Staphylococci; Staphylococcus aureus;* and *Enterococcus* spp.		406		Culture-based	Kirby–Bauer disc diffusion	CLSI	Food handlers	26.5	406	Food vending stations	Human
Asamoah et al. [28]	Multicenter (Accra and	2022	Cross-sectional	Jan 2017-Dec 2021.	Resistance to three or more antibiotic drug classes.	*Escherichia coli; Klebsiella* spp.; *Proteus* spp.; *Pseudomonas* spp.;	*Klebsiella* spp.	20,010	68.6%	Culture-based	Kirby–Bauer disc diffusion	CLSI	Patients with Urinary Tract	38.8	328,073	MDS Lancet Laboratories	Human

	Ashanti)					and *Acinetobacter* spp.							Infections				
Sah et al. [8]	Western	2022	Cross-sectional	Oct 2020-Feb 2021	n.a	*Escherichia coli; Klebsiella* spp.; *Enterobacter* spp.; *Citrobacter* spp.; *Salmonella* spp.; *Proteus* spp.; and *Pseudomonas aeruginosa*	*Escherichia coli*	258	48.6%	Culture-based; and standard biochemical tests	Kirby–Bauer disc diffusion	CLSI	Clinical isolate	28	807	Hospitals	Human
Gnimatin et al. [7]	Northern	2022	Retrospective Cross-sectional	Jun 2018-May 2022	Resistant to at least one antibiotic agent in any three or more antibacterial categories	*Acinetobacter* spp.; *Enterobacter* spp.; *Klebsiella* spp.; *Moraxella* spp.; *Escherichia coli; Pseudomonas spp.; Staphylococcus* spp.; *Sphingomonas* spp. *Shewanella* spp.; *Providencia* spp.; *Photobacterium* spp.; *Gardnerella* spp.; *Raoultella* spp. *Raoultella* spp.; *Salmonella* spp.; and *Streptococcus* spp.	*Staphylococcus* spp.	1,222	41.6%	Culture-based; standard biochemical tests; and API 20E system (bioMérieux SA, Marcy l'Etoile, France)	Kirby–Bauer disc diffusion	CLSI	Clinical isolate	41	1,222	Tamale Zonal Public Health Reference Laboratory	Human
Dekker et al. [1]	Ashanti	2016	n.a	May 2007-August 2012	Resistance to at least three groups of antibiotics or being an MRSA	*Staphylococcus aureus*	*Staphylococcus aureus*	9,834	35.7%	Culture-based; standard biochemical tests; and Multiplex polymerase chain reaction (PCR)	VITEK 2 system (AST 603 cards, bioMerieux, France)	EUCAST	Patients of all age groups	3	9,834	St. Michael's Hospital (SMH)	Human
Labi et al. [24]	Greater Accra	2014	Retrospective	Jan 2010-Dec 2013	In vitro non-susceptibility to ≥1 agent in ≥3 antimicrobial categories	*Salmonella* spp.	*Salmonella* spp.	181	44.8%	BACTEC 9240 blood culture system; Crystal Enteric/Nonfermenter identification system®	Kirby-Bauer disc diffusion	CLSI	Clinical isolate	9.5	2,768	Korle Bu Teaching Hospital (KBTH).	Human
Abana et al. [11]	Greater Accra	2019	n.a	Oct 2015-Jan 2016	n.a	*Vibrio cholerae*	*Vibrio cholerae*	n.a	97%	Culture-based; standard biochemical tests; V. cholerae O1 polyvalent antiserum (Remel Europe Ltd., UK); and PCR analysis	Kirby-Bauer disc diffusion	CLSI	Clinical isolates	n.a	51	Korle Bu Teaching Hospital (KBTH)	Human

Krumkamp et al. [10]	Ashanti	2020	n.a	n.a		*Escherichia coli; Pseudomonas aeruginosa; Staphylococcus aureus; Klebsiella pneumoniae; Streptococcus* spp.; and *Candida* spp.				Culture-based; standard biochemical tests; MALDI Biotyper standard method (Bruker Daltonics, Bremen, Germany); and qPCR assay	VITEK 2 system	EUCAST	Patients with chronically infected wounds	54	105	Kumasi Centre for Collaborative Research in Tropical Medicine, Ghana	Human
Janssen et al. [16]	Western	2018	Prospective	Mar–Jul 2014	n.a	*Staphylococcus aureus; Enterococcus faecalis; Proteus mirabilis; Escherichia coli; Klebsiella pneumoniae; Enterobacter cloacae; Pseudomonas aeruginosa;* and *Acinetobacter baumannii*	n.a	32	n.a	Culture-based; standard biochemical tests; and MALDI Biotyper 3.0 (Bruker Daltonics, Bremen, Germany).	VITEK 2 (bioMérieux, Marcy-l'Étoile, France)	EUCAST version 4.0	Hospital-acquired wound infection patients	40.1	67	St. Martin de Porres Hospital	Human
Karika et al. [21]	Northern	2022	Prospective cross-sectional	Apr 2018-Sep 2018	Resistance of isolates to 3 or more classes of antibiotics.	*Escherichia coli; Staphylococcus aureus; Staphylococcus* spp.; *Klebsiella* spp.; *Serratia* spp.; *Proteus* spp. and *Streptococcus* spp.	*Escherichia coli*	74	87.9% - 92.7%	Culture-based; and standard biochemical tests	Kirby-Bauer disk diffusion method.	CLSI	Cases of Urinary Tract Infections	39.18	219	Tamale Teaching Hospital (TTH) and the Tamale Central Hospital (TCH)	Human
Asare et al. [22]	Central	2022	Retrospective cross-sectional	Jan 2013-Dec 2015	n.a	*Staphylococcus* spp.; *Neisseria gonorrhoeae; Escherichia coli; Citrobacter* spp.; and *Klebsiella* spp.	n.a	1,515	n.a	Culture-based; and standard biochemical tests	Kirby-Bauer disk diffusion method	n.a	Patients attending the outpatient department	25-26	1,515	University of Cape Coast hospital	Human
Inusah et al. [3]	Upper East	2021	Retrospective	Jan 2018-July 2020	Resistant to at least one agent in three or more antimicrobial categories	*Escherichia coli; Klebsiella* spp.; *Proteus* spp.; *Pseudomonas* spp.; Citrobacter spp.; *Enterobacter* spp.; *Moraxella* spp.; *Morganella* spp.; *Providencia* spp.; *Salmonella* spp.;	*Escherichia coli*	800	99.6%	Culture-based; and standard biochemical tests	Kirby–Bauer disc diffusion method	n.a	General population	n.a	800	Bolgatanga Regional hospital, Ghana	Human

						Serratia spp.; *Shigella* spp.; *Neisseria* spp.; *Staphylococcus* spp.; and *Streptococcus* spp.											
Attram et al. [4]	Multicenter (Accra and Western)	2019	n.a	Sep 2012-Dec 2015	n.a	*Neisseria gonorrhoeae*	n.a	44	n.a	Culture-based; standard biochemical tests; and API-NH (Biomerieux, Marcy l'Etoile, France) test	Kirby–Bauer disc diffusion method	CLSI	General population	n.a	990	Five health facilities	Human
Bekoe et al. [6]	Greater Accra	2022	n.a	n.a	n.a	Coagulase-negative staphylococci; and *Staphylococcus* spp.	n.a	63	n.a	Culture-based; standard biochemical tests; and MALDI-TOF/MS (Bruker Biotyper system, Microflex LT/SH MS, Roskilde, Denmark)	n.a	EUCAST	Healthy volunteers	n.a	401	Korle-Gonno and Dodowa	Human
Quansah et al. [9]	Multicenter (Upper East, Ashanti and Central)	2019	n.a	Oct 2017-Mar 2018	n.a	*Klebsiella* spp.	n.a	139	n.a	Culture-based; standard biochemical tests; and DNA	n.a	CLSI	General population	n.a	139	Health facilities	Human
Agyepong et al. [13]	Ashanti	2018	n.a	Feb-Aug 2015	Resistant to at least one agent in three or more antibiotic classes.	*Klebsiella pneumoniae; Acinetobacter baumannii; Pseudomonas aeruginosa; Enterobacter* spp.; *Escherichia coli; Yersinia* spp.; *Proteus mirabilis; Pasteurella* spp.; *Chromobacterium violaceum; Salmonella enterica; Vibrio* spp.; *Citrobacter koseri; Pantoea* spp.; *Serratia* spp.; *Providencia* spp.; *Burkholderia*	*Pseudomonas aeruginosa*	200	89.5%	Culture-based; and standard biochemical tests	Vitek-2 (Biomerieux, France) Automated Systems	n.a	In-patients and out-patients.	35.95	200	Komfo Anokye Teaching Hospital (KATH)	Human

						cepacia; Aeromonas spp.; *Cadecea lapagei;* and *Sphingomonas paucimobilis*											
Deininger et al. [14]	Volta	2022	Retrospective	Oct 2019–Dec 2021	n.a	*Escherichia coli; Candida* spp.; *Klebsiella* spp.; *Citrobacter* spp.; *Proteus* spp.; *Salmonella* spp.; *Staphylococcus* spp.; and *Pseudomonas* spp.	n.a	131	n.a	Culture-based; and standard biochemical tests	Kirby–Bauer disc diffusion method	CLSI	Inpatient or outpatient	40	348	Margret Marquart Catholic Hospital (MMCH)	Human
Andoh et al. [17]	Ashanti	2017	n.a	n.a	Resistant to three or more classes of antimicrobials	*Salmonella* spp.	*Salmonella* spp.	137	42.3%	Culture-based; and standard biochemical tests	n.a	EUCAST	Patients with suspected septicemia or diarrhea	n.a	137	Two hospitals	Human
Mohammed et al. [18]	Greater Accra	2018	Retrospective	Jan-Dec 2016	n.a	*Staphylococcus* spp.; *Staphylococcus aureus;* and *Escherichia coli*	n.a	10,237	n.a	Culture-based; and standard biochemical tests	Kirby-Bauer disk diffusion method	CLSI	General population	n.a	10,237	Three hospitals and three private diagnostic service laboratories.	Human
Codjoe et al. [25]	Multicenter (Accra, Western, Ashanti and Volta)	2019	Prospective	Sept 2012-Sep 2014	n.a	*Pseudomonas aeruginosa;* Acinetobacter spp.; *Escherichia coli; Pseudomonas* spp.; *Klebsiella pneumoniae; Enterobacter* spp.; *Cronobacter* spp.; *Providencia* spp.; *Shigella* spp.; and *Sphingomonas* spp.	n.a	3,840	n.a	Culture-based; and standard biochemical tests	Kirby-Bauer disk diffusion method	CLSI	General population	n.a	3,840	Hospitals	Human
Agbeko et al. [29]	Multicenter (Western and Central)	2022	n.a	Oct 2019-Feb 2020	n.a	*Serratia* spp.; *Escherichia coli; Edwardsiella* spp.; *Citrobacter* spp.; *Shigella* spp.; *Staphylococcus aureus;* and *Staphylococcus* spp.	n.a	145	n.a	Culture-based; standard biochemical tests; and DNA	Kirby-Bauer disk diffusion method	CLSI	Fish	n.a	145	Nine fish farms	Animal

Adinortey et al. [30]	Central	2020	n.a	n.a	n.a	*Citrobacter* spp.; *Klebsiella pneumoniae; Escherichia coli; Serratia* spp. and *Proteus mirabilis*	*Escherichia coli*	n.a	100%	Culture-based; and standard biochemical tests	Kirby-Bauer disk diffusion method	CLSI	Fish	n.a	n.a	Six small-to-medium scale fish farms	Animal
Mensah et al. [31]	Greater Accra	2022	Cross-sectional	Jan-Mar 2020	Resistance to at least 2 classes of antibiotics	*Escherichia coli*	*Escherichia coli*	75	57.1-62.5%	Culture-based; standard biochemical tests; and MALDITOF-MS	Kirby-Bauer disk diffusion method	CLSI	Fecal and chicken samples	n.a	75	Selected Farms and Retail Outlets	Animal
Boamah et al. [32]	Multicenter (Brong Ahafo, Ashanti, and Greater Accra	2017	n.a	n.a	Simultaneous resistance to three or more antibiotics	Coagulase-negative *Staphylococci*	Coagulase-negative *Staphylococci*	368	19.14%	Culture-based; standard biochemical tests; and MALDITOF-MS	Kirby-Bauer disk diffusion method	EUCAST	Fecal and chicken samples	n.a	368	400 poultry farms	Animal
Labi et al. [33]	Greater Accra	2022	Cross-sectional	Jan-Mar 2022	Resistance to three antimicrobial classes	*Escherichia coli; Enterobacter* spp.; and *Klebsiella pneumoniae*	*Escherichia coli*	197	23%	Culture-based; and standard biochemical tests	Kirby-Bauer disk diffusion method	CLSI	Rectal swabs from pigs	4 months	200	20 pig farms	Animal
Andoh et al. [34]	Multicenter (Accra and Kumasi	2016	Cross-sectional	n.a	Resistance to ≥3 classes of antimicrobials	*Salmonella* spp.	*Salmonella* spp.	94	40·4%	Culture-based; and standard biochemical tests	Kirby-Bauer disk diffusion method	EUCAST	Fecal and chicken samples	n.a	200	20 poultry farms from Accra and 55 farms from Kumasi	Animal
Adzitey et al. [35]	Northern	2020	n.a	n.a	Resistance to ≥1 agent in >3 antibiotic classes	*Escherichia coli; Citrobacter* spp.; *Enterobacter* spp.; *Klebsiella* spp.	*Escherichia coli*	189	68.33%	Culture-based; and standard biochemical tests	Kirby-Bauer disk diffusion method	CLSI	Meat samples from beef, chevon, mutton, chicken, and guinea fowl	n.a	225	Tamale Metropolis	Animal
Paintsil et al. [36]	Ashanti	2022	Cross-sectional	Mar 2019-Jul 2020	Resistance to three or more antimicrobials of any substance group	*Campylobacter* spp.	*Campylobacter* spp.	322	23.3%	Culture-based; standard biochemical tests; and MALDI-TOF MS	Kirby-Bauer disk diffusion method	EUCAST	Poultry and livestock faecal samples	n.a	1,585	Commercial and smallholder farms	Animal
Dsani et al. [37]	Greater Accra	2020	Cross-sectional	Jan 2019-Jun 2019	Resistance to three or more classes of	*Escherichia coli*	*Escherichia coli*	98	22%	Culture-based; and MALDI-TOF MS	Kirby-Bauer disk diffusion	n.a.	Beef, chevon and	n.a	205	Slaughterhouses	Animal

					antimicrobial agents						method		mutton samples			
Karikari et al. [38]	Ashanti	2017	n.a	Mar 2013-Feb 2014	Resistance to three or more antibiotics	*Campylobacter* spp.	*Campylobacter jejuni*	241	66.6%	Culture-based; standard biochemical tests; and API CAMPY system (bioMérieux, Marcy l'Etoile, France)	Kirby-Bauer disk diffusion method	EUCAST and CLSI	Faeces and Carcasses of Healthy Livestock	n.a	938	Kumasi Abattoir	Animal
Asuming-Bediako et al. [39]	Greater Accra	2022	n.a	Febr 2019-Jan 2020	n.a	*Campylobacter* spp.	*Campylobacter jejuni*	153	26.9%	Culture-based; standard biochemical tests; and MALDI-TOF MS	n.a	n.a	Raw retail chicken meat	n.a	400	Metropolitan Accra	Animal
Baah et al. [40]	Accra	2022	Cross-sectional	n.a	Resistance to three or more antibiotic classes	*Escherichia coli; Aeromonas hydrophila; Vibrio cholerae;* and *Klebsiella pneumoniae*	*Escherichia coli*	558	14.9%	Culture-based; standard biochemical tests; API 20E (Biomerieux SA, Marcy-l'Étoile, France), and MALDI-TOF (Bruker Daltonics GmbH & Co., Bremen, Germany, MBT Compass IVD Ver. 4.2.100)	Kirby-Bauer disk diffusion method	CLSI	Meats (beef, goat meat, and chicken)	n.a	270	Meat vending shops	Animal
Eibach et al. [41]	Ashanti	2018	n.a	May-Dec 2015	n.a	*Escherichia coli;* and *Klebsiella pneumoniae*	n.a	71	n.a	Culture-based; standard biochemical tests; API 20E (bioMérieux, Marcy L'Etoile, France); and MALDI-TOF mass spectrometry (Bruker, Billerica, USA)	n.a	EUCAST	Local and imported poultry meat	n.a	200	94 stores and markets	Animal
Dekker et al. [42]	Ashanti	2019	Cross-sectional	May-Dec 2015	Resistance to three classes of antibiotics	*Salmonella* spp.; *Campylobacter* spp.; and *Arcobacter* spp.	*Campylobacter* spp.	92	20%	Culture-based; standard biochemical tests; and MALDI-TOF MS	n.a	EUCAST	Frozen and freshmeat	n.a	200	Poultry industry and imports	Animal
Saba et al. [43]	Northern	2022	n.a	Mar-Apr 2016	Resistance to three or more antibiotics	*Staphylococcus aureus;* and *Escherichia coli*	n.a	78	n.a	Culture-based; and standard biochemical tests	n.a	EUCAST	Mobile phones of health personnel	n.a	220	Three major government hospitals in the	Environmental

																Tamale Metropolis of Ghana	
Odonkor et al. [44]	Multicenter (Accra and Eastern)	2022	n.a	n.a	n.a	*Acinetobacter* spp.; *Bacillus* spp.; *Citrobacter* spp.; *Enterobacter* spp.; *Enterococcus* spp.; *Escherichia coli; Klebsiella* spp.; *Proteus* spp.; *Providencia* spp.; *Pseudomonas aeruginosa; Salmonella* spp.; *Staphylococcus aureus; Staphylococcus* spp.; *Streptococcus* spp.; and *Vibrio spp.*	*Staphylococcus aureus*	110	59.6%	Selective media methods and the analytical profile index (API)	Kirby-Bauer disk diffusion method	CLSI	Boreholes, dams, hand-dug wells	n.a	67	Seven communities	Environmental
Adomako et al. [45]	Greater Accra	2021	Cross-sectional	Jan–Jun 2018	n.a	*Escherichia coli; Aeromonas hydrophila* and *Pseudomonas aeruginos*	*n.a*	150	n.a	Culture-based; and standard biochemical tests	Kirby-Bauer disk diffusion method	CLSI	Wastewater and surface water samples	n.a	150	Legon Sewage Treatment plant	Environmental
Saba et al. [46]	Northern	2017	n.a	Feb-Mar 2015	Resistant to three or more antibiotics	*Staphylococcus aureus*	*Staphylococcus aureus*	47	19%	Culture-based; and standard biochemical tests	n.a	CLSI	Door handles and other points of contact	n.a	120	Tamale Teaching Hospital, Tamale Central Hospital and Tamale West Hospital	Environmental
Ahmed et al. [47]	Accra	2022	Cross-sectional	Dec 2021-Mar 2022.	Resistance to three or more classes of antibiotics.	*Escherichia coli;* and *Pseudomonas aeruginosa*	*Escherichia coli*	317	58.3%	Culture-based; and MALDI-TOF MS (Bruker MALDI Biotyper, Billerica, MA, USA).	Kirby-Bauer disk diffusion method	CLSI	Potable water samples	n.a	542	CSIR–Water Research Institute,	Environmental
Addae-Nuku et al. [48]	Accra	2022	Longitudinal	Oct-Dec 2021	Resistance to 3 or more classes of antimicrobial drugs.	*Escherichia coli; Klebsiella pneumoniae; Citrobacter* spp.; *Alcaligenes faecalis; Pseudomonas* spp.	*Escherichia coli*	294	28%	Culture-based; standard biochemical tests; and MALDI-TOF	Kirby–Bauer disc diffusion	CLSI	Hospital Wastewater		288	Korle Bu Teaching Hospital (KBTH).	Environmental

Table S4: Medically important bacteria isolated in the studies.

Bacteria Isolate	Human Studies (28) n (%)	Animal Studies (14) n (%)	Environmental Studies (6) n (%)	References
Acinetobacter spp.	7 (25)	1 (7.14)	2 (33.33)	[13,15,16,18,20,25,28] [40] [44,48]
Campylobacter spp.		4 (28.57)		[36,38,39,42]
Candida spp.	2 (7.14)			[14,22]
Citrobacter spp.	9 (32.14)	3 (21.43)	2 (33.33)	[3,5,8,13,14,18,19,22,27] [29,30,40] [44,48]
Coagulase-negative Staphylococci	5 (18.86)	1 (7.14)		[5,6,19,23,27] [32]
E. coli	19 (67.86)	8 (57.14)	5 (83.33)	[2,3,7,8,10,12–16,18–23,26–28] [29–31,33,35,37,40,41][43–45,47,48]
Enterobacter spp.	9 (32.14)	4 (28.57)	1 (16.67)	[3,8,13,15,16,18,19,21,27] [29,30,33,40] [44]
Enterococcus spp.	6 (21.43)		1 (16.67)	[10,16,18,23,27,28] [44]
Klebsiella spp.	18 (64.29)	5 (35.71)	2 (33.3)	[2,3,7–10,13–16,18–21,23,26–28] [29,30,33,40,41] [44,48]
Neisseria spp.	4 (14.29)			[3–5,22]
Proteus spp.	12 (42.46)	3 (21.43)	1 (16.67)	[3,8,10,13,14,16,18,19,21,22,27,28] [29,30,40] [44]
Providencia spp.	6 (21.43)	1 (7.14)	1 (16.67)	[3,7,13,15,18,19] [40] [44]
Pseudomonas spp.	14 (50)	1 (7.14)	4 (66.67)	[3,7,8,13–16,18–20,23,25,27,28][40] [44,45,47,48]
S. aureus	10 (35.71)		3 (50)	[1,5,10,16,18,19,21–23,27] [43,44,46]
Salmonella spp.	12 (42.86)	5 (35.71)	1 (16.67)	[2,3,8,13,14,17–22,24] [29,30,34,40,42] [44]

Shigella	2 (7.14)	3 (21.43)		[2,3] [29,30,40]
Staphylococcus spp.	4 (14.29)	1 (7.14)		[3,7,14,18] [29]
Streptococcus spp.	6 (21.43)	1 (7.14)	1 (16.67)	[3,5,10,18,21,23] [29] [44]
Vibrio spp.	3 (10.71)	1 (7.14)		[2,11,13] [40]

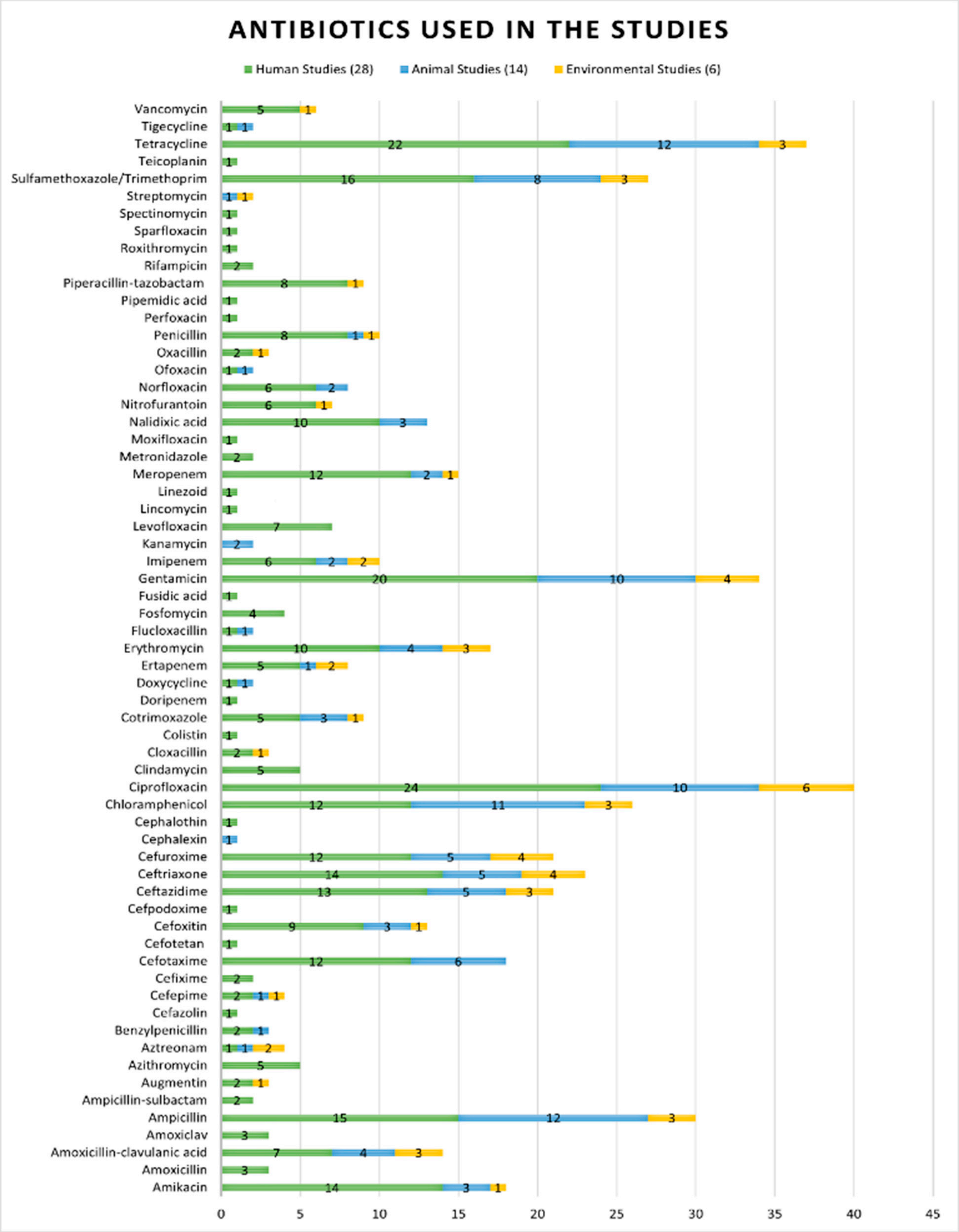

Figure S1. Antibiotic used in the studies.

References

1. Dekker D, Wolters M, Mertens E, Boahen KG, Krumkamp R, Eibach D, et al. Antibiotic resistance and clonal diversity of invasive Staphylococcus aureus in the rural Ashanti Region, Ghana. BMC Infect Dis [Internet]. 2016 Nov 29;16(1):720. Available from: https://pubmed.ncbi.nlm.nih.gov/27899074

2. Afum T, Asandem DA, Asare P, Asante-Poku A, Mensah GI, Musah AB, et al. Diarrhea-Causing Bacteria and Their Antibiotic Resistance Patterns Among Diarrhea Patients From Ghana. Front Microbiol [Internet]. 2022 May 19;13:894319. Available from: https://pubmed.ncbi.nlm.nih.gov/35663873

3. Inusah A, Quansah E, Fosu K, Dadzie I. Resistance Status of Bacteria from a Health Facility in Ghana: A Retrospective Study. J Pathog [Internet]. 2021 Mar 4;2021:6648247. Available from: https://pubmed.ncbi.nlm.nih.gov/33747569

4. Attram N, Agbodzi B, Dela H, Behene E, Nyarko EO, Kyei NNA, et al. Antimicrobial resistance (AMR) and molecular characterization of Neisseria gonorrhoeae in Ghana, 2012-2015. PLoS One [Internet]. 2019 Oct 10;14(10):e0223598–e0223598. Available from: https://pubmed.ncbi.nlm.nih.gov/31600300

5. Sampane-Donkor E, Badoe EV, Annan JA, Nii-Trebi NI. Colonisation of antibiotic resistant bacteria in a cohort of HIV infected children in Ghana. Pan Afr Med J [Internet]. 2017 Feb 2 [cited 2023 Oct 15];26:1937–8688. Available from: /pmc/articles/PMC5398869/

6. Bekoe SO, Hane-Weijman S, Trads SL, Orman E, Opintan J, Hansen M, et al. Reservoir of Antibiotic Residues and Resistant Coagulase Negative Staphylococci in a Healthy Population in the Greater Accra Region, Ghana. Antibiotics (Basel) [Internet]. 2022 Jan 17;11(1):119. Available from: https://pubmed.ncbi.nlm.nih.gov/35052997

7. Gnimatin JP, Weyori EW, Agossou SM, Adokiya MN. Bacterial infections epidemiology and factors associated with multidrug resistance in the northern region of Ghana. Sci Rep [Internet]. 2022 Dec 21;12(1):22069. Available from: https://pubmed.ncbi.nlm.nih.gov/36543904

8. Sah AK, Feglo PK. Plasmid-mediated quinolone resistance determinants in clinical bacterial pathogens isolated from the Western Region of Ghana: a cross-sectional study. Pan Afr Med J [Internet]. 2022 Dec 27;43:207. Available from: https://pubmed.ncbi.nlm.nih.gov/36942137

9. Quansah E, Amoah Barnie P, Omane Acheampong D, Obiri-Yeboah D, Odarkor Mills R, Asmah E, et al. Geographical Distribution of β-Lactam Resistance among Klebsiella spp. from Selected Health Facilities in Ghana. Trop Med Infect Dis [Internet]. 2019 Sep 3;4(3):117. Available from: https://pubmed.ncbi.nlm.nih.gov/31484298

10. Krumkamp R, Oppong K, Hogan B, Strauss R, Frickmann H, Wiafe-Akenten C, et al. Spectrum of antibiotic resistant bacteria and fungi isolated from chronically infected wounds in a rural district hospital in Ghana. PLoS One [Internet]. 2020 Aug 7;15(8):e0237263–e0237263. Available from: https://pubmed.ncbi.nlm.nih.gov/32764812

11. Abana D, Gyamfi E, Dogbe M, Opoku G, Opare D, Boateng G, et al. Investigating the virulence genes and antibiotic susceptibility patterns of Vibrio cholerae O1 in environmental and clinical isolates in Accra, Ghana. BMC Infect Dis [Internet]. 2019 Jan 21;19(1):76. Available from: https://pubmed.ncbi.nlm.nih.gov/30665342

12. Deku JG, Duedu KO, Ativi E, Kpene GE, Feglo PK. Occurrence and distribution of extended-spectrum β-lactamase in clinical Escherichia coli isolates at Ho Teaching Hospital in Ghana. Ghana Med J [Internet]. 2021 Dec;55(4):298–307. Available from: https://pubmed.ncbi.nlm.nih.gov/35957938

13. Agyepong N, Govinden U, Owusu-Ofori A, Essack SY. Multidrug-resistant gram-negative bacterial infections in a teaching hospital in Ghana. Antimicrob Resist Infect Control [Internet]. 2018 Mar 9;7:37. Available from: https://pubmed.ncbi.nlm.nih.gov/29541448

14. Deininger S, Gründler T, Deininger SHM, Lütcke K, Lütcke H, Agbesi J, et al. The Antimicrobial Resistance (AMR) Rates of Uropathogens in a Rural Western African Area-A Retrospective Single-Center Study from Kpando, Ghana. Antibiotics (Basel) [Internet]. 2022 Dec 13;11(12):1808. Available from: https://pubmed.ncbi.nlm.nih.gov/36551465

15. Osei MM, Dayie NTKD, Azaglo GSK, Tettey EY, Nartey ET, Fenny AP, et al. Alarming Levels of Multidrug Resistance in Aerobic Gram-Negative Bacilli Isolated from the Nasopharynx of Healthy Under-Five Children in Accra, Ghana. Int J Environ Res Public Health [Internet]. 2022 Sep 1;19(17):10927. Available from: https://pubmed.ncbi.nlm.nih.gov/36078645

16. Janssen H, Janssen I, Cooper P, Kainyah C, Pellio T, Quintel M, et al. Antimicrobial-Resistant Bacteria in Infected Wounds, Ghana, 2014(1). Emerg Infect Dis [Internet]. 2018 May;24(5):916–9. Available from: https://pubmed.ncbi.nlm.nih.gov/29664368

17. Andoh LA, Ahmed S, Olsen JE, Obiri-Danso K, Newman MJ, Opintan JA, et al. Prevalence and characterization of Salmonella among humans in Ghana. Trop Med Health [Internet]. 2017 Feb 10;45:3. Available from: https://pubmed.ncbi.nlm.nih.gov/28194090

18. Mohammed J, Hounmanou YMG, Thomsen LE. Antimicrobial resistance among clinically relevant bacterial isolates in Accra: a retrospective study. BMC Res Notes [Internet]. 2018 Apr 25;11(1):254. Available from: https://pubmed.ncbi.nlm.nih.gov/29695265

19. Asafo-Adjei K, Mensah JE, Labi AK, Dayie NTKD, Donkor ES. Urinary Tract Infections among Bladder Outlet Obstruction Patients in Accra, Ghana: Aetiology, Antibiotic Resistance, and Risk Factors. Diseases [Internet]. 2018 Jul 19;6(3):65. Available from: https://pubmed.ncbi.nlm.nih.gov/30029549

20. Donkor ES, Muhsen K, Johnson SAM, Kotey FCN, Dayie NTKD, Tetteh-Quarcoo PB, et al. Multicenter Surveillance of Antimicrobial Resistance among Gram-Negative Bacteria Isolated from Bloodstream Infections in Ghana. Antibiotics (Basel) [Internet]. 2023 Jan 27;12(2):255. Available from: https://pubmed.ncbi.nlm.nih.gov/36830166

21. Karikari AB, Saba CK, Yamik DY. Reported Cases of Urinary Tract Infections and the Susceptibility of Uropathogens from Hospitals in Northern Ghana. Microbiol Insights [Internet]. 2022 Jun

24;15:11786361221106108–11786361221106108. Available from: https://pubmed.ncbi.nlm.nih.gov/35769634

22. Asare KK, Amoah S, Coomson Jr CA, Banson C, Yaro D, Mbata J, et al. Antibiotic-resistant pathogenic bacterial isolates from patients attending the outpatient department of university of Cape Coast hospital, Ghana: A retrospective study between 2013-2015. PLOS global public health [Internet]. 2022 May 16;2(5):e0000417–e0000417. Available from: https://pubmed.ncbi.nlm.nih.gov/36962199

23. Omenako KA, Enimil A, Marfo AFA, Timire C, Chinnakali P, Fenny AP, et al. Pattern of Antimicrobial Susceptibility and Antimicrobial Treatment of Neonates Admitted with Suspected Sepsis in a Teaching Hospital in Ghana, 2021. Int J Environ Res Public Health [Internet]. 2022 Oct 10;19(19):12968. Available from: https://pubmed.ncbi.nlm.nih.gov/36232262

24. Labi AK, Obeng-Nkrumah N, Addison NO, Donkor ES. Salmonella blood stream infections in a tertiary care setting in Ghana. BMC Infect Dis [Internet]. 2014 Dec 21;14:3857. Available from: https://pubmed.ncbi.nlm.nih.gov/25528352

25. Codjoe FS, Donkor ES, Smith TJ, Miller K. Phenotypic and Genotypic Characterization of Carbapenem-Resistant Gram-Negative Bacilli Pathogens from Hospitals in Ghana. Microbial Drug Resistance [Internet]. 2019;25(10):1449–57. Available from: http://dx.doi.org/10.1089/mdr.2018.0278

26. Dwomoh FP, Kotey FCN, Dayie NTKD, Osei MM, Amoa-Owusu F, Bannah V, et al. Phenotypic and genotypic detection of carbapenemase-producing Escherichia coli and Klebsiella pneumoniae in Accra, Ghana. PLoS One [Internet]. 2022 Dec 30;17(12):e0279715–e0279715. Available from: https://pubmed.ncbi.nlm.nih.gov/36584159

27. Vicar EK, Alo DB, Koyiri VC, Opare-Asamoah K, Obeng-Bempong M, Mensah GI. Carriage of Antibiotic Resistant Bacteria and Associated Factors Among Food Handlers in Tamale Metropolis, Ghana: Implications for Food Safety. Microbiol Insights [Internet]. 2023 Jan 28;16:11786361221150696–11786361221150696. Available from: https://pubmed.ncbi.nlm.nih.gov/36726578

28. Asamoah B, Labi AK, Gupte HA, Davtyan H, Peprah GM, Adu-Gyan F, et al. High Resistance to Antibiotics Recommended in Standard Treatment Guidelines in Ghana: A Cross-Sectional Study of Antimicrobial Resistance Patterns in Patients with Urinary Tract Infections between 2017-2021. Int J Environ Res Public Health [Internet]. 2022 Dec 9;19(24):16556. Available from: https://pubmed.ncbi.nlm.nih.gov/36554436

29. Agbeko R, Aheto DW, Asante DKA, Asare NK, Boateng AA, Adinortey CA. Identification of molecular determinants of antibiotic resistance in some fish farms of Ghana. Heliyon [Internet]. 2022 Aug 30;8(9):e10431–e10431. Available from: https://pubmed.ncbi.nlm.nih.gov/36097488

30. Adinortey CA, Aheto DW, Boateng AA, Agbeko R. Multiple Antibiotic Resistance-Coliform Bacteria in Some Selected Fish Farms of the Central Region of Ghana. Scientifica (Cairo) [Internet]. 2020 Dec 4;2020:6641461. Available from: https://pubmed.ncbi.nlm.nih.gov/33376622

31. Mensah GI, Adjei VY, Vicar EK, Atsu PS, Blavo DL, Johnson SAM, et al. Safety of Retailed Poultry: Analysis of Antibiotic Resistance in Escherichia coli From Raw Chicken and Poultry Fecal Matter From Selected Farms and Retail Outlets in Accra, Ghana. Microbiol Insights [Internet]. 2022 Apr 29;15:11786361221093278–11786361221093278. Available from: https://pubmed.ncbi.nlm.nih.gov/35521227

32. Boamah VE, Agyare C, Odoi H, Adu F, Gbedema SY, Dalsgaard A. Prevalence and antibiotic resistance of coagulase-negative Staphylococci isolated from poultry farms in three regions of Ghana. Infect Drug Resist [Internet]. 2017 Jun 13;10:175–83. Available from: https://pubmed.ncbi.nlm.nih.gov/28652785

33. Ohene Larbi R, Adeapena W, Ayim-Akonor M, Ansa EDO, Tweya H, Terry RF, et al. Antimicrobial, Multi-Drug and Colistin Resistance in Enterobacteriaceae in Healthy Pigs in the Greater Accra Region of Ghana, 2022: A Cross-Sectional Study. Int J Environ Res Public Health [Internet]. 2022 Aug 22;19(16):10449. Available from: https://pubmed.ncbi.nlm.nih.gov/36012083

34. Andoh LA, Dalsgaard A, Obiri-Danso K, Newman MJ, Barco L, Olsen JE. Prevalence and antimicrobial resistance of Salmonella serovars isolated from poultry in Ghana. Epidemiol Infect [Internet]. 2016/06/23. 2016 Nov;144(15):3288–99. Available from: https://pubmed.ncbi.nlm.nih.gov/27334298

35. Adzitey F, Assoah-Peprah P, Teye GA, Somboro AM, Kumalo HM, Amoako DG. Prevalence and Antimicrobial Resistance of Escherichia coli Isolated from Various Meat Types in the Tamale Metropolis of Ghana. Int J Food Sci [Internet]. 2020 Nov 12;2020:8877196. Available from: https://pubmed.ncbi.nlm.nih.gov/33274191

36. Paintsil EK, Ofori LA, Akenten CW, Zautner AE, Mbwana J, Jaeger A, et al. Antibiotic-resistant Campylobacter coli and Campylobacter jejuni in commercial and smallholder farm animals in the Asante Akim North Municipality of Ghana. Front Microbiol [Internet]. 2022 Nov 4;13:983047. Available from: https://pubmed.ncbi.nlm.nih.gov/36406391

37. Dsani E, Afari EA, Danso-Appiah A, Kenu E, Kaburi BB, Egyir B. Antimicrobial resistance and molecular detection of extended spectrum β-lactamase producing Escherichia coli isolates from raw meat in Greater Accra region, Ghana. BMC Microbiol [Internet]. 2020 Aug 14;20(1):253. Available from: https://pubmed.ncbi.nlm.nih.gov/32795260

38. Karikari AB, Obiri-Danso K, Frimpong EH, Krogfelt KA. Antibiotic Resistance of Campylobacter Recovered from Faeces and Carcasses of Healthy Livestock. Biomed Res Int [Internet]. 2017/01/18. 2017;2017:4091856. Available from: https://pubmed.ncbi.nlm.nih.gov/28194411

39. Asuming-Bediako N, Kunadu APH, Jordan D, Abraham S, Habib I. Prevalence and antimicrobial susceptibility pattern of Campylobacter jejuni in raw retail chicken meat in Metropolitan Accra, Ghana. Int J Food Microbiol [Internet]. 2022;376:109760. Available from: http://dx.doi.org/10.1016/j.ijfoodmicro.2022.109760

40. Baah DA, Kotey FCN, Dayie NTKD, Codjoe FS, Tetteh-Quarcoo PB, Donkor ES. Multidrug-Resistant Gram-Negative Bacteria Contaminating Raw Meat Sold in Accra, Ghana. Pathogens [Internet]. 2022 Dec 11;11(12):1517. Available from: https://pubmed.ncbi.nlm.nih.gov/36558851

41. Eibach D, Dekker D, Gyau Boahen K, Wiafe Akenten C, Sarpong N, Belmar Campos C, et al. Extended-spectrum beta-lactamase-producing Escherichia coli and Klebsiella pneumoniae in local and imported poultry meat in Ghana. Vet Microbiol [Internet]. 2018;217:7–12. Available from: http://dx.doi.org/10.1016/j.vetmic.2018.02.023

42. Dekker D, Eibach D, Boahen KG, Akenten CW, Pfeifer Y, Zautner AE, et al. Fluoroquinolone-Resistant Salmonella enterica, Campylobacter spp., and Arcobacter butzleri from Local and Imported Poultry Meat in Kumasi, Ghana. Foodborne Pathog Dis [Internet]. 2019/03/23. 2019 May;16(5):352–8. Available from: https://pubmed.ncbi.nlm.nih.gov/30907631

43. Saba CKS, Naa-Inour F, Kpordze SW. Antibiotic resistance pattern of methicillin resistant *staphylococcus aureus* and *Escherichia coli* from mobile phones of healthcare workers in public hospitals in Ghana. Pan African Medical Journal [Internet]. 2022;41. Available from: http://dx.doi.org/10.11604/pamj.2022.41.259.29281

44. Odonkor ST, Simpson SV, Morales Medina WR, Fahrenfeld NL. Antibiotic-Resistant Bacteria and Resistance Genes in Isolates from Ghanaian Drinking Water Sources. J Environ Public Health [Internet]. 2022 Oct 6;2022:2850165. Available from: https://pubmed.ncbi.nlm.nih.gov/36246472

45. Adomako LAB, Yirenya-Tawiah D, Nukpezah D, Abrahamya A, Labi AK, Grigoryan R, et al. Reduced Bacterial Counts from a Sewage Treatment Plant but Increased Counts and Antibiotic Resistance in the Recipient Stream in Accra, Ghana-A Cross-Sectional Study. Trop Med Infect Dis [Internet]. 2021 May 14;6(2):79. Available from: https://pubmed.ncbi.nlm.nih.gov/34068850

46. Saba CKS, Amenyona JK, Kpordze SW. Prevalence and pattern of antibiotic resistance of Staphylococcus aureus isolated from door handles and other points of contact in public hospitals in Ghana. Antimicrob Resist Infect Control [Internet]. 2017 May 10;6:44. Available from: https://pubmed.ncbi.nlm.nih.gov/28491294

47. Ahmed H, Zolfo M, Williams A, Ashubwe-Jalemba J, Tweya H, Adeapena W, et al. Antibiotic-Resistant Bacteria in Drinking Water from the Greater Accra Region, Ghana: A Cross-Sectional Study, December 2021-March 2022. Int J Environ Res Public Health [Internet]. 2022 Sep 28;19(19):12300. Available from: https://pubmed.ncbi.nlm.nih.gov/36231603

48. Addae-Nuku DS, Kotey FC, Dayie NT, Osei MM, Tette EM, Debrah P, et al. Multidrug-Resistant Bacteria in Hospital Wastewater of the Korle Bu Teaching Hospital in Accra, Ghana. Environ Health Insights [Internet]. 2022 Oct 22;16:11786302221130612–11786302221130612. Available from: https://pubmed.ncbi.nlm.nih.gov/36311334
